# Performance of Alzheimer Disease Plasma Biomarkers in Patients With Prion Diseases

**DOI:** 10.1212/WNL.0000000000214712

**Published:** 2026-07-13

**Authors:** Thomas Coysh, Rhiannon Laban, Elena Veleva, Amanda J. Heslegrave, Lee Darwent, Leah Holm-Mercer, Tze How Mok, Melanie Sarah Hart, Michael Peter Lunn, Ashvini Keshavan, Jonathan M. Schott, Zane Jaunmuktane, Sebastian Brandner, Fernando Gonzalez-Ortiz, Kaj Blennow, Henrik Zetterberg, John Collinge, Simon Mead

**Affiliations:** 1 MRC Prion Unit at University College London (UCL), UCL Institute of Prion Diseases, London, UK;; 2 NHS National Prion Clinic, National Hospital for Neurology and Neurosurgery, University College London Hospitals NHS Foundation Trust, London, UK;; 3 United Kingdom Dementia Research Institute at UCL, London, UK;; 4 Department of Neurodegenerative Disease, Queen Square Institute of Neurology, UCL, London, UK;; 5 NHS Neuroimmunology and CSF Laboratory, Queen Square Institute of Neurology, UCL, London, UK;; 6 Department of Neuroinflammation, UCL, London, UK;; 7 Centre for Neuromuscular Disease, National Hospital for Neurology and Neurosurgery, University College London Hospitals NHS Foundation Trust, London, UK;; 8 Division of Neuropathology, National Hospital for Neurology and Neurosurgery, University College London Hospitals NHS Foundation Trust, London, UK;; 9 Department of Clinical and Movement Neurosciences and Queen Square Brain Bank for Neurological Disorders, Queen Square Institute of Neurology, UCL, London, UK;; 10 Department of Psychiatry and Neurochemistry, Sahlgrenska Academy at the University of Gothenburg, Sweden;; 11 Clinical Neurochemistry Laboratory, Sahlgrenska University Hospital, Sweden;; 12 Paris Brain Institute, ICM, Pitié-Salpêtrière Hospital, Sorbonne University, Paris, France;; 13 Neurodegenerative Disorder Research Center, Division of Life Sciences and Medicine, and Department of Neurology, Institute on Aging and Brain Disorders, University of Science and Technology of China and First Affiliated Hospital of USTC, Hefei, China; and; 14 Hong Kong Center for Neurodegenerative Diseases, InnoHK, China.

## Abstract

**Background and Objectives:**

Prion diseases can mimic Alzheimer disease (AD) at presentation. Alzheimer's Association AD diagnostic criteria suggest that a single abnormal highly specific plasma biomarker (including p-tau217) is sufficient for a biological diagnosis. We investigated the performance of AD plasma biomarkers in distinguishing AD and prion diseases.

**Methods:**

We examined plasma biomarker data from patients with prion disease from a prospective cohort study recruited through the UK National Prion Clinic. Prion diseases were diagnosed clinically or with autopsy confirmation, and AD was diagnosed clinically with CSF biomarker confirmation. Plasma p-tau217, p-tau181, Aβ42/40 ratio, brain-derived tau (BD-tau), neurofilament light chain (NfL), and glial fibrillary acid protein (GFAP) were measured using Simoa. Median biomarker values in different groups were compared with Kruskal-Wallis test, and area under the receiver operating characteristic curve was used to compare accuracy in distinguishing prion diseases from sporadic AD (sAD). Lumipulse p-tau217 and NfL were measured in a validation study in a different laboratory.

**Results:**

In the main study, we analyzed 345 samples from 278 individuals (mean age 58 [SD 13.5], 48.2% female), including 204 with prion diseases (121 sporadic Creutzfeldt-Jakob disease [CJD], 11 iatrogenic CJD, 9 variant CJD, 47 slow-progressing inherited prion disease (IPD) and 16 fast-progressing IPD), 33 with AD, and 41 healthy controls. For discriminating prion disease without AD copathology from sAD, none of p-tau217 (area under the curve [AUC] [95% CI] 0.605 [0.486–0.724]), p-tau181 (AUC 0.554 [0.446–0.661]), or GFAP (AUC 0.514 [0.389–0.640]) performed well. Aβ42/40 discriminated moderately (AUC 0.770 [0.684–0.856]). NfL/p-tau217 ratio (AUC 0.996 [0.987–1.000]), NfL (AUC 0.988 [0.974–1.000]), BD-tau/p-tau217 ratio (AUC 0.963 [0.929–0.996]), and BD-tau (AUC 0.934 [0.890–0.978]) discriminated very well. In an independent validation study, consecutive samples were analyzed from 32 patients with sAD and 35 patients with sporadic Creutzfeldt-Jakob disease (mean age 65.0 [SD 6.4], 56.7% female). NfL/p-tau217 again discriminated almost perfectly (AUC 0.986 [95% CI 0.966–1.000]).

**Discussion:**

Plasma p-tau217 and p-tau181 are increased in both AD and prion diseases (regardless of burden of AD copathology). Diagnosing AD with a single abnormal p-tau plasma biomarker risks misdiagnosing prion diseases as AD. Plasma NfL/p-tau217 discriminates near-perfectly and could act as a flag to suspect prion diseases where this is a diagnostic possibility.

**Classification of Evidence:**

This study provides Class II evidence that plasma NfL/p-tau217 discriminates patients with CJD from those with AD.

## Introduction

The development of ultrasensitive plasma biomarker assays that measure proteins involved in Alzheimer disease (AD) pathobiology including phosphorylated tau (p-tau) and β-amyloid (Aβ) has created optimism that these tests could revolutionize AD diagnosis by enabling noninvasive, cost-efficient biomarker confirmation of underlying AD neuropathologic change (ADNC).^1,2^ Alzheimer's Association diagnostic criteria advocate separating clinical and biological diagnosis. They suggest a single abnormal “Core 1” biomarker of p-tau or Aβ (including accurate plasma biomarkers such as p-tau217) would be sufficient to establish a diagnosis, even in asymptomatic patients.^1^ However, little is known about how these tests perform in prion diseases, which can mimic AD clinically, and early studies show contrasting results.^3,4^

Human prion diseases are fatal transmissible neurodegenerative diseases with a shared mechanism of formation of amyloid fibrillar assemblies of misfolded cellular prion protein, which propagate by elongation and fission.^5^ They cause approximately 1 in 5,000 deaths in countries where they are monitored well.^6^ Sporadic Creutzfeldt-Jakob disease (CJD), the most common human prion disease, is thought to arise from spontaneous formation of prions. Inherited prion diseases (IPDs) occur because of prion protein gene (*PRNP*) coding alterations. Acquired prion diseases arise from transmission of bovine spongiform encephalopathy prions (variant CJD) or human-human transmission through medical procedures (iatrogenic CJD).

Prion diseases including sporadic Creutzfeldt-Jakob disease (sCJD) are usually associated with rapid neurocognitive decline, but initial presentation as a pure cognitive syndrome is common,^7^ and in such cases, progression is typically slower.^8^ Diagnosis and referral to specialist services is often delayed until other symptoms and signs emerge by which time patients are severely impaired,^9^ despite advanced diagnostic tests (MRI and real-time quaking-induced conversion) which otherwise permit early and accurate diagnosis.^10^ This presents a problem as rationally designed therapeutics against prion disease enter clinical testing,^11,12^ because meaningful treatment benefits are most likely in the early stages.

This study aimed to determine false positive rates for AD plasma biomarker tests in prion diseases and explore mitigations for blood-based biomarker diagnosis of neurodegenerative diseases. Plasma p-tau species are of particular concern because, although reported as highly accurate plasma biomarkers of AD, we predicted they would also be elevated in prion diseases, because hyperphosphorylated tau deposition is a neuropathologic feature of prion diseases^13,14^ and plasma and CSF total tau, p-tau181, and p-tau217 have, to varying extents, been reported to be elevated in prion diseases.^3,4,13,15-19^ In this study, we evaluated plasma p-tau217, p-tau181, Aβ42/40 ratio, neurofilament light chain (NfL), brain-derived tau (BD-tau), and glial fibrillary acid protein (GFAP) from healthy controls, patients with AD, and patients with prion disease in a prospective natural history cohort including all types of prion disease, using postmortem cases to directly assess AD copathology. Our research questions were as follows: how do AD plasma biomarkers perform in discriminating AD and prion diseases? and what are the relationships between these biomarkers and prion disease progression measured directly using validated clinimetrics?^9,20^

## Methods

### Participants and Plasma Samples

Individuals were eligible if they had enrolled in the National Prion Monitoring Cohort study^9^ or the PRION-1 trial,^21^ collectively known as “the Cohort,” and had an EDTA plasma sample available. These included patients diagnosed by contemporary diagnostic criteria^22^ with sCJD, variant CJD (vCJD), iatrogenic Creutzfeldt-Jakob disease (iCJD) (secondary to cadaveric human growth hormone exposure), slow IPD (6-OPRI, P102L, A117V, Y163X, P157X, E146G, and T107I *PRNP* alterations), fast IPD (E200K and D178N *PRNP* alterations); 3 patients diagnosed with iatrogenic AD (iAD)^23^; and healthy controls (friends and relatives of patients with prion disease without known neurologic disease, excluding those with or at risk of carrying *PRNP* mutations). These individuals overlap with participants in previous publications, but all biomarker data are new.^9,16,21,23,24^ These patients were referred to the National Prion Clinic, through UK prion disease clinical or surveillance pathways or because of being at-risk of developing prion disease, and had blood collected between 2003 and 2023 for the main study, and between 2024 and 2025 for the validation study. Before 2015, most patients seen by the National Prion Clinic had postmortem examination of the brain by consultant neuropathologists with expertise in prion disease. Prion disease with significant AD copathology was defined as postmortem confirmed prion disease with ADNC intermediate or higher, or Braak stage III or above, or significant AD copathology reported.^25^ Patients with sporadic AD (i.e., late- or early-onset AD without a gene alteration causing familial AD) diagnosed according to contemporary diagnostic criteria with CSF biomarker confirmation of diagnosis,^26^ as defined by published cut-points,^27^ at the National Hospital for Neurology and Neurosurgery (NHNN) Cognitive Disorders Clinic were also included. In the main study, the latest sample was used for per-individual analysis, to minimize time to postmortem. In the validation study, the first sample was used, from sequentially recruited patients.

### Measurement of Biomarker Concentrations

EDTA plasma was analyzed blinded to diagnosis. A Quanterix Simoa HD-X analyzer at the UK Dementia Research Institute (DRI) Fluid Biomarker Laboratory was used for p-tau181 using the P-tau181 Version 2.1 Advantage kit and for Aβ42, Aβ40, NfL, and GFAP using the Neurology 4-Plex E kit according to the manufacturer's protocol.^28^ Intraplate and interplate coefficients of variation were ≤15% and ≤12%, respectively. A Quanterix Simoa HD-X analyzer at the Clinical Neurochemistry Laboratory at Sahlgrenska University Hospital was used for p-tau217 using ALZpath P-tau217 Version 2 kit,^29^ and for BD-tau using a homebrew assay using TauJ5.H3 (Bioventix) as capture antibody and Tau12 (BioLegend, #SIG-39416) as detection antibody, following published methods.^30^ Two quality control samples were run in duplicate at the start and end of each run and demonstrated repeatability <5.5% and intermediate precision ≤9.0%. For the validation study, EDTA plasma was tested with Lumipulse G NfL and p-tau217 chemiluminescent enzyme immunoassay on Fujirebio G1200 analyzer in the NHNN Neuroimmunology and CSF laboratory according to the manufacturer's protocol. Inter-assay and intra-assay coefficients of variation were <4%.

### Prospective Clinical Data

Patients with prion disease had clinical scales data collected prospectively at enrollment and at follow-up appointments, scheduled between fortnightly and annually,^9,21^ according to rate of progression. The Medical Research Council (MRC) Prion Disease Rating Scale, “MRC Scale,” provides a validated interval-level functional outcome measure in sCJD, running from 20 (no significant impairment) to 0 (bedbound, mute, unaware of surroundings and unable to swallow).^9^ For statistical analysis, the MRC Scale can be transformed from its raw 20 point scale to a 100 point logit-equivalent scale and repeated measures in patients modeled by linear mixed effects model, which provides a measure of rate of functional decline (MRC Scale slope).^20^ MRC Scale slope requires *PRNP* codon 129 genotype and excludes those with severe functional impairment (MRC Scale <5) at enrollment.

### Statistical Analysis

Biomarker values were positively skewed, so median values were compared with Kruskal-Wallis test or Mann-Whitney *U* test. Post-hoc pairwise comparisons between diagnostic groups for each biomarker were performed using Dunn test with Bonferroni correction to control family-wise error. We did not apply additional correction across biomarkers, as each assay was analyzed as a distinct, biologically motivated hypothesis rather than as part of a discovery set. Biomarker values were age-adjusted to age 60 years for NfL, GFAP, and Aβ42/40, using previously published methods.^31^ Aβ42/40 was not age-adjusted for the purposes of testing to discriminate diagnostic groups, as age-adjustment removed its ability to discriminate sporadic AD (sAD) from healthy controls (eTable 1), similar to previous reports on the effect of plasma Aβ42/40 adjustment for age and other confounders,^32^ so if used clinically it would have to be used without age adjustment. Multiple linear regression of log_10_ (age-adjusted) biomarker was used to assess their relationships with disease stage or rate of decline with *PRNP* codon 129 genotype as a covariate. Linear mixed effects modeling of longitudinal change in log_10_(biomarker) with disease progression in patients with serial blood samples was done in 2 different ways: using fixed effects of days before death or MRC Scale. All models also included fixed effects for age, and random effects for slope and intercept to account for variation between individuals. All regression models were performed with log(biomarker) and log(biomarker) standardized by expressing as z-score, to ensure valid comparisons. Summaries of models are provided in the supplementary material. Scatter plot followed by Spearman rank correlation (r_s_) was used to assess monotonic relationship between other non-normally distributed or ordinal data. Statistical analysis and data visualization was performed using Microsoft Excel v16.77.1, GraphPad Prism v10.2.3, and Stata v17.0.

### Standard Protocol Approvals, Registrations, and Patient Consents

All participants provided written informed research consent. Patients with prion disease and iAD and healthy controls were enrolled in the cohort (Scotland A Research Ethics Committee 05/MRE00/63) or the development of new diagnostic tests for prion disease study (Queen Square Research Ethics Committee 03/N022). Patients with sAD were enrolled in the CSF collection in patients with neurodegenerative diseases study (Queen Square Research Ethics Committee 12/LO/1504).

### Data Availability

Data including biomarker values can be made available upon request but will require a data transfer agreement.

## Results

### Comparison of Plasma AD Biomarkers Between Diagnostic Groups

**Figure 1:**
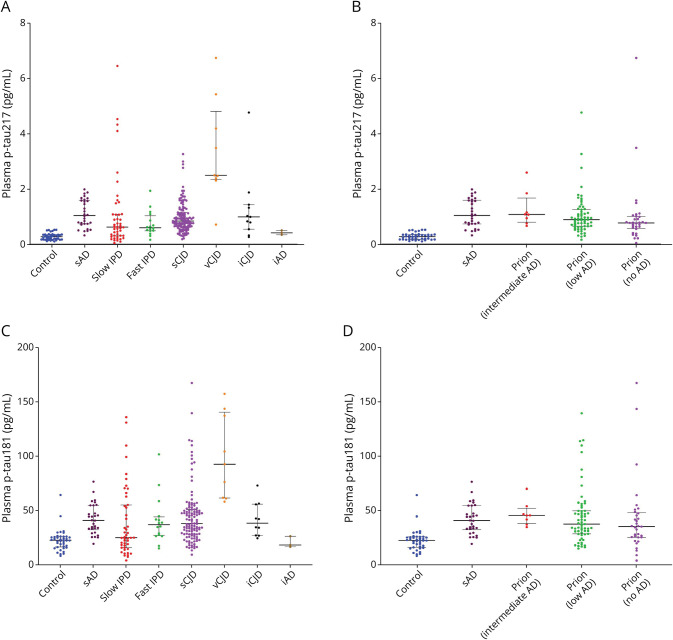
Plasma P-Tau217 and P-Tau181 in Different Diagnostic Groups (A) Plasma p-tau217 and (C) plasma p-tau181 for each individual in different diagnostic groups with median and interquartile range underlaid. (B) Plasma p-tau217 and (D) plasma p-tau181 values for each individual in sporadic Alzheimer disease (AD), healthy control, and prion cases with autopsy assessment of AD copathology, with median and interquartile range underlaid. fast IPD = fast-progressing inherited prion disease; iAD = iatrogenic Alzheimer disease; iCJD = iatrogenic Creutzfeldt-Jakob disease; sAD = sporadic Alzheimer disease; sCJD = sporadic Creutzfeldt-Jakob disease; slow IPD = slow-progressing inherited prion disease; vCJD = variant Creutzfeldt-Jakob disease.

Summary statistics and estimates of precision (interquartile range [IQR]) for biomarkers tested, and demographic details, are displayed in Table 1. Plasma p-tau217 showed significant differences across diagnostic categories (Figure 1A; Kruskal-Wallis, *p* < 0.0001). Relative to healthy controls, plasma p-tau217 was elevated in sCJD (median fold change [FC] 3.11 [IQR 2.37–4.24], Dunn test, *p* < 0.0001); slow IPD (FC 2.30 [1.11–4.01], *p* < 0.0001); fast IPD (FC 2.20 [1.79–3.83], *p* = 0.0087), vCJD (FC 9.22 [8.67–17.78], *p* < 0.0001), iCJD (FC 3.67 [2.00–5.30], *p* < 0.0001), and sAD (FC 3.85 [2.71–5.88], *p* < 0.0001). P-tau217 was not significantly different in any prion disease relative to sAD (all *p* > 0.05). In keeping with the strong correlation between p-tau217 and p-tau181 (*r*_*s*_ [272] = 0.85 [95% CI 0.81–0.88]), p-tau181 results were similar. P-tau181 was elevated relative to controls in all prion diseases with smaller FC, ranging from 1.11 (slow IPD) to 4.12 (vCJD), and p-tau181 was elevated relative to sAD in vCJD but not in other prion diseases (Figure 1C). Patients with vCJD exhibited the greatest elevation relative to sAD for both p-tau217 (FC 2.39 [IQR 2.25–4.62]) and p-tau181 (FC 2.27 [1.51–3.37]). All patients with vCJD with neuropathologic assessment had no ADNC and marked PrP^Sc^-related p-tau deposition.^14^

**Table 1:** Summary of Participants in the Main Study and Summary Statistics for Plasma P-Tau181, P-Tau217, Aβ42/40 Ratio, NfL, GFAP, and BD-tau

Diagnosis	n	Mean age (SD)	F/M	*PRNP* c129				Autopsy assessment of ADNC	Plasma p-tau181^a^ (pg/mL)			Plasma p-tau217^b^ (pg/mL)			Plasma Aβ42/40 ratio^c^			Plasma NfL (pg/mL)^d^			Plasma GFAP (pg/mL)^e^			Plasma BD-tau (pg/mL)^e^		
				MM	MV	VV	Not tested		Min	Median (Q1 - Q3)	Max	Min	Median (Q1 - Q3)	Max	Min	Median (Q1 - Q3)	Max	Min	Median (Q1 - Q3)	Max	Min	Median (Q1 - Q3)	Max	Min	Median (Q1 - Q3)	Max
Healthy control	41	53.6 (13.3)	18/23	NA	NA	NA	NA	NA	7.98	22.39 (16.17–25.39)	64.06	0.10	0.27 (0.17–0.35)	0.52	0.0348	0.0621 (0.0546–0.0775)	0.0908	5.01	12.50 (10.84–15.82)	53.93	28.16	67.94 (54.02–99.12)	309.98	0.71	1.21 (1.05–1.43)	3.64
Sporadic AD	30	64.4 (5.9)	16/14	NA	NA	NA	NA	NA	19.06	40.62 (32.06–54.44)	76.25	0.32	1.04 (0.73–1.59)	1.98	0.0400	0.0555 (0.0513–0.0585)	0.0837	9.94	19.19 (15.00–28.13)	62.17	92.45	344.79 (254.24–816.93)	1662.75	0.80	1.33 (1.04–1.71)	3.00
Slow IPD^f^	47	50.4 (12.1)	24/23	23	19	4	1	10	3.78	24.80 (16.54–54.81)	135.61	0.04	0.62 (0.30–1.08)	6.44	0.0449	0.0650 (0.0564–0.0781)	0.330	8.06	53.37 (27.61–101.15)	900.06	56.17	307.67 (182.65–536.27)	1460.27	0.25	1.63 (1.12–3.47)	25.50
Fast IPD^g^	16	62.2 (11.1)	9/7	11	4	1	0	7	14.93	36.58 (26.48–43.75)	101.42	0.16	0.60 (0.48–1.03)	1.93	0.0473	0.0757 (0.0606–0.0875)	0.161	36.22	119.93 (85.08–273.09)	485.16	98.42	177.22 (120.07–355.09)	986.40	0.98	8.89 (4.07–30.65)	113.00
Sporadic CJD	121	65.0 (10.2)	62/59	43	42	25	11	82	9.10	37.77 (27.91–50.63)	167.12	0.18	0.84 (0.64–1.15)	3.26	0.00911	0.0679 (0.0586–0.0796)	0.271	34.22	185.42 (112.95–293.93)	2,666.27	89.64	468.27 (253.14–825.12)	2,680.61	1.45	11.60 (4.91–27.80)	252.00
Variant CJD	9	29.5 (10.6)	2/7	8	1	0	0	2	57.88	92.30 (61.15–136.94)	157.07	0.71	2.49 (2.34–4.80)	6.73	0.0561	0.0784 (0.0598–0.105)	0.141	130.90	175.63 (138.59–339.39)	526.48	142.99	578.56 (306.76–2,290.41)	4,778.93	2.15	4.25 (3.54–5.48)	6.89
Iatrogenic CJD	11	44.9 (6.9)	2/9	4	6	1	0	3	24.24	38.08 (26.74–55.55)	72.69	0.26	0.99 (0.54–1.43)	4.76	0.0486	0.0674 (0.0514–0.0836)	0.129	92.29	238.34 (143.89–497.70)	758.37	114.84	232.22 (134.17–448.86)	1530.09	1.80	4.07 (1.84–17.00)	57.10
Iatrogenic AD	3	55.0 (1.7)	1/2	NA	NA	NA	NA	1	16.34	17.94 (16.34–26.04)	26.04	0.34	0.41 (0.34–0.50)	0.50	0.0394	0.0602 (0.0394–0.0678)	0.0678	6.80	131.16 (6.80–148.45)	148.45	76.53	148.94 (76.53–172.58)	172.58	1.11	1.75 (1.11–3.21)	3.21

Abbreviations: AD = Alzheimer’s disease; ADNC = Alzheimer disease neuropathologic change; BD- tau = brain-derived tau; CJD = Creutzfeldt-Jakob disease; GFAP = glial fibrillary acid protein; iCJD = iatrogenic Creutzfeldt-Jakob disease; IPD = inherited prion disease; MM = methionine/methionine; MV = methionine/valine; NA = not applicable; NfL = neurofilament light chain; sAD = sporadic AD; sCJD = sporadic CJD; VV = valine/valine.

The plasma sample used in per individual analysis (displayed in this table) was the latest one available.

a Not age-adjusted; 1 sCJD, 1 iCJD, and 1 healthy control could not be analyzed.

b Not age-adjusted; 1 healthy control, 1 sAD, 1 slow IPD, 1 fast IPD, and 3 sCJD could not be analyzed.

c Age-adjusted; 1 healthy control, 1 sAD, and 2 sCJD could not be analyzed.

d Age-adjusted; 1 sAD and 1 sCJD could not be analyzed.

e Not age-adjusted, 1 slow IPD could not be analyzed.

f 6-OPRI (11), A117 (2), E146G (2), P102L (25), P157X (2), T107I (2), Y163X (3).

g D178N (6), E200K (10).

Elevation of plasma p-tau217 and p-tau181 in prion diseases was not explained by AD copathology (Figure 1, B and D). P-tau217 was elevated compared to healthy controls in prion diseases with no ADNC (FC 2.85 [IQR 2.08–3.72]; Dunn test, *p* < 0.0001); in prion diseases with low ADNC (FC 3.30 [2.41–4.63], *p* < 0.0001); and in prion diseases with intermediate ADNC (FC 3.98 [2.95–6.18], *p* < 0.0001). There were no significant differences in p-tau217 between sAD and prion disease with no ADNC (FC 0.74 [0.54–0.97], *p* = 0.497), low ADNC (FC 0.86 [0.63–1.20], *p* = 1.00), or intermediate ADNC (FC 1.03 [0.77–1.60], *p* = 1.00) or between prion disease groups with no, low, or intermediate ADNC (all *p* > 0.7). Similar results were observed for p-tau181 (Figure 1D).

Receiver operating characteristic (ROC) curve analysis revealed that despite excellent discrimination of sAD from controls (eTable 1), neither p-tau217 nor p-tau181 could discriminate sAD from prion diseases without significant AD copathology (Table 2, eFigure 1). To assess whether the problem of p-tau217's poor performance as a single biomarker can be simply rectified by applying the AT (N) biomarker system in blood, we tested Aβ42/40 ratio as a measure of Aβ deposition (A), p-tau217 as a measure of pathologic tau (T), and NfL as a measure of neurodegeneration (N),^26^ with cutoffs defined by Youden method^33^ for sAD vs control (eTable 1). About 31/118 (26%) of sCJD and 50/198 (25%) of all prion disease cases would be misclassified as A + T + N+.

**Table 2:** ROC Curve Analysis for Discrimination of Prion Disease Without Significant AD Copathology From Sporadic AD Using Plasma Biomarkers

	Age-adjusted NfL/p-tau217	Age-adjusted NfL/p-tau181	Age-adjusted NfL	BD-tau	Aβ42/40	P-tau217/Aβ40	P-tau217/Aβ42	P-tau217^a^	P-tau181^a^	Αge-adjusted GFAP^a^
AUC (95% CI)	0.996 (0.987–1.000)	0.995 (0.988–1.000)	0.988 (0.974–1.000)	0.934 (0.890–0.978)	0.770 (0.684–0.856)	0.755 (0.669–0.843)	0.718 (0.623–0.812)	0.605 (0.486–0.724)	0.554 (0.446–0.661)	0.514 (0.389–0.640)
Cutoff (Youden^33^ method)	>58.57	>1.087	>64.89 pg/mL	>3.025 pg/mL	>0.0616	>0.0209	>0.3529	<1.180 pg/mL	<24.51 pg/mL	>348.40 pg/mL
Sensitivity (95% CI)	93.68 (86.90–97.07)	96.84 (91.12–99.14)	90.53 (82.97–94.94)	84.21 (75.57–90.19)	68.09 (58.11–76.64)	56.84 (46.81–66.34)	56.99 (46.85–66.58)	78.95 (69.71–85.94)	19.79 (13.05–28.86)	64.58 (54.62–73.42)
Specificity (95% CI)	100.00 (88.30–100.00)	100.00 (88.30–100.00)	100.00 (88.30–100.00)	100.00 (88.65–100.00)	93.10 (78.04–98.77)	86.21 (69.44–94.50)	82.76 (65.45–92.40)	43.33 (27.38–60.80)	96.67 (83.33–99.83)	55.17 (37.55–71.59)
Sensitivity applying cutoff defined above to prion disease diagnostic groups vs sAD^b^, (%)										
sCJD (n = 121)	97.50	98.32	94.96	92.56	67.80	57.50	59.16	76.03	18.33	64.17
vCJD (n = 9)	66.67	100.00	100.00	88.89	66.67	100.00	77.78	11.11	0.00	66.67
iCJD (n = 11)	100.00	100.00	100.00	54.55	54.55	54.55	36.36	63.64	10.00	45.45
Slow IPD (n = 47)	71.74	74.47	48.94	28.26	58.70	32.60	26.67	78.26	46.81	44.68
Fast IPD (n = 16)	93.75	87.50	81.25	81.25	73.33	75.00	73.33	87.50	12.50	31.25

Abbreviations: AD = Alzheimer’s disease; AUC = area under the curve; BD-tau = brain‐derived tau; GFAP = glial fibrillary acid protein; iCJD = iatrogenic Creutzfeldt‐Jakob disease; IPD = inherited prion disease; NfL = neurofilament light chain; ROC = receiver operating characteristic; sAD = sporadic AD; sCJD = sporadic CJD; vCJD = variant CJD.

a GFAP, p-tau217, and p-tau181 are no better than chance at discriminating prion disease without significant AD copathology vs sAD (*p* > 0.05) so have no discriminatory value.

b Prion disease groups including all patients, not just those with postmortem examination for AD copathology. Specificities are not displayed as they are the same as reported in the top section of the table because the sAD group is the same.

**Figure 2:**
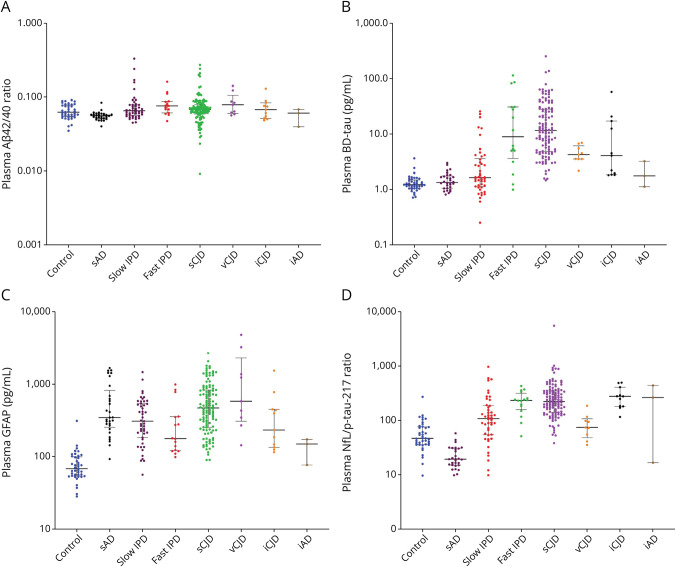
Plasma Aβ42/40 Ratio, Brain-Derived Tau, GFAP, and NfL/P-Tau217 Ratio in Different Diagnostic Groups (A) Aβ42/40 ratio, (B) brain-derived tau (BD-tau), (C) age-adjusted GFAP, (D) ratio of age-adjusted NfL to p-tau217 plotted with log_10_ y axis in different diagnostic groups with median and interquartile range underlaid. fast IPD = fast-progressing inherited prion disease; GFAP = glial fibrillary acid protein; iAD = iatrogenic Alzheimer disease; iCJD = iatrogenic Creutzfeldt-Jakob disease; NfL = neurofilament light chain; sAD = sporadic Alzheimer disease; sCJD = sporadic Creutzfeldt-Jakob disease; slow IPD = slow-progressing inherited prion disease; vCJD = variant Creutzfeldt-Jakob disease.

**Figure 3:**
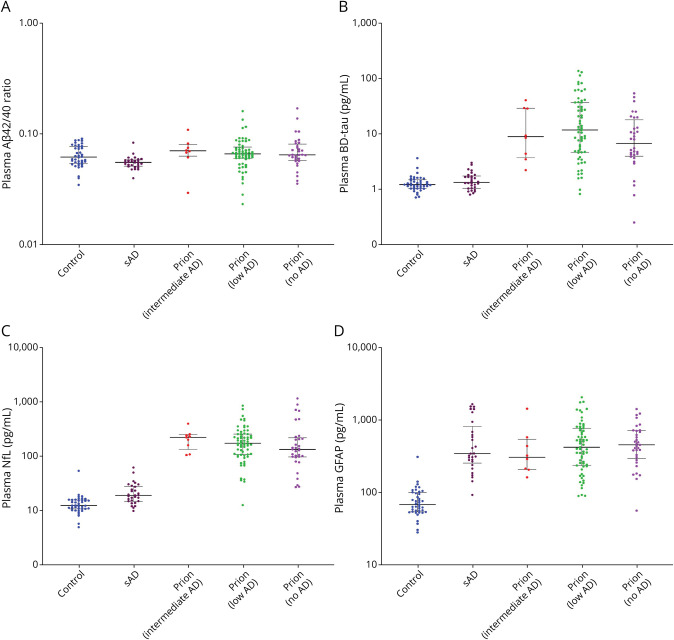
Plasma Aβ42/40 Ratio, Brain-Derived Tau, NfL, and GFAP in Patients With Prion Disease With Postmortem Assessment of AD Neuropathologic Change, Sporadic AD, and Healthy Controls Plots of (A) Aβ42/40, (B) brain-derived tau (BD-tau), (C) age-adjusted NfL, and (D) age-adjusted GFAP in healthy controls, patients with sporadic AD (sAD), and patients with prion disease with intermediate, low, and no AD neuropathologic change, with median and interquartile range underlaid. AD = Alzheimer’s disease; GFAP = glial fibrillary acid protein; NfL = neurofilament light chain.

Next, we assessed the performance of other plasma AD biomarkers Aβ42/40, BD-tau, NfL, and GFAP in distinguishing diagnostic categories. Aβ42/40 showed significant differences across diagnostic categories (Figure 2A; Kruskal-Wallis test, *p* = 0.0002) with a significantly decreased Aβ42/40 ratio in sAD compared with sCJD (FC 0.82 [IQR 0.76–0.86], Dunn test, *p* = 0.0004), slow IPD (FC 0.85 [0.79–0.90], *p* = 0.0049), fast IPD (FC 0.73 [0.68–0.77], *p* = 0.0010), and vCJD (FC 0.71 [0.66–0.75], *p* = 0.0159) but no significant difference when compared with other diagnostic categories, including healthy controls (FC 0.89 [0.83–0.94], *p* = 0.147). Aβ42/40 was lower in sAD compared with prion disease with no ADNC (FC 0.85 [0.79–0.90], *p* = 0.0023), low ADNC (FC 0.84 [0.78–0.88], *p* = 0.0004), and intermediate ADNC (FC 0.79 [0.73–0.83], *p* = 0.0315) and compared with controls (FC 0.89 [0.83–0.94], *p* = 0.0357). There was no significant difference in Aβ42/40 between prion disease cases with different levels of ADNC (Figure 3A, all *p* = 1.00).

There was no significant correlation between age-adjusted Aβ42/40 and ADNC in prion disease cases (*r*_*s*_ [100] = 0.18, 95% CI –0.02 to 0.37, *p =* 0.0733) or sCJD cases only (*r*_*s*_ [79] = 0.01, 95% CI –0.11 to 0.33, *p* = 0.315). These results suggest that in prion diseases, Aβ42/40 ratio does not act as a biomarker of ADNC. Indeed, both Aβ42 and Aβ40 had a similar pattern of significantly decreased values in prion diseases relative to both sAD and controls (eFigure 2). In sCJD, relative to sAD, Aβ42 was decreased 0.47-fold (IQR 0.30- to 0.66-fold, Dunn test *p* < 0.0001) and Aβ40 was decreased 0.43-fold (0.27- to 0.71-fold, *p* < 0.0001).

BD-tau showed significant differences across diagnostic categories (Figure 2B; Kruskal-Wallis test, *p* < 0.0001). BD-tau was elevated relative to sAD in sCJD (FC 8.7 [IQR 3.69–20.90], Dunn test *p* < 0.0001), fast IPD (FC 6.68 [3.06–23.05], *p* < 0.0001), vCJD (FC 3.20 [2.66–4.12], *p* = 0.139), and iCJD (FC 3.06 [1.38 to 12.78], *p* = 0.0252). BD-tau was not significantly elevated in slow IPD compared with sAD (FC 1.22 [0.68 to 2.61], *p* = 1.00) or in sAD compared with control (FC 1.09 [0.86–1.41], *p* = 1.00). Results for NfL were similar (eFigure 3), except for that NfL was elevated in all prion disease groups compared with sAD, including slow IPD.

Considering prion disease cases with assessment of ADNC (Figure 3B), BD-tau was elevated compared with sAD in prion disease cases with no ADNC (FC 5.06 [3.26–9.97], *p* < 0.0001), low ADNC FC 8.83 [3.49–27.70], *p* < 0.0001), and intermediate ADNC (FC 6.70 [2.79–27.84], *p* = 0.0017), but there was no significant difference in BD-tau between prion disease cases with different levels of ADNC (all *p* = 1.00). Results for NfL were similar (Figure 3C). BD-tau was strongly correlated with NfL (*r*_*s*_ [275] = 0.83, 95% CI 0.79–0.87, *p* < 0.0001) in keeping with its proposed status as a biomarker of neurodegeneration,^30^ whereas it was only moderately correlated with p-tau217 (*r*_*s*_ [275] = 0.52, 95% CI 0.43–0.61, *p* < 0.0001) and p-tau181 (*r*_*s*_ [272] = 0.47, 95% CI 0.37–0.56, *p* < 0.0001). BD-tau could not discriminate sAD from controls (eTable 1).

GFAP showed significant differences across diagnostic categories (Figure 2C, Kruskal-Wallis test, *p* < 0.0001), with GFAP elevated relative to controls in all prion disease groups (sCJD FC 6.89 [IQR 3.73–12.14], vCJD FC 8.52 [4.51–33.71] and slow IPD FC 4.53 [2.69–7.89], Dunn test all *p* < 0.0001; fast IPD FC 2.61 [1.77–5.23], *p* = 0.0062; iCJD FC 3.42 [1.97– 6.61], *p* = 0.0038) and in sAD (FC 5.07 [3.74–12.02], *p* < 0.0001). There was no significant difference in GFAP between sAD and any prion diagnostic groups (all *p* = 1.00) and no significant difference in GFAP between prion disease cases with different levels of ADNC (all *p* = 1.00, eAppendix 1).

As plasma p-tau species are the preeminent plasma biomarkers of sAD,^2,34-37^ but cannot distinguish sAD from prion disease, plasma NfL/p-tau ratio (Figure 2D) was tested as a means to discriminate prion diseases and sAD, using highly elevated NfL levels seen in all prion diseases, because of intense neurodegeneration, to distinguish the elevated p-tau seen in both sAD and prion diseases. ROC analysis was performed for discrimination of prion disease without significant AD copathology from sAD (Table 2, eFigure 4). NfL/p-tau217 had the greatest area under the ROC curve (AUC = 0.996, 95% CI 0.987–1.000), followed by NfL/p-tau181 (AUC = 0.995, 95% CI 0.988–1.00), NfL (AUC = 0.988, 95% CI 0.974–1.000), BD-tau (AUC = 0.934, 95% CI 0.890–0.978), and Aβ42/40 (AUC = 0.770, 95% CI 0.684–0.856). BD-tau/p-tau217 ratio discriminated slightly better than BD-tau alone (AUC = 0.963, 95% CI 0.929– 0.996) but less well than NfL. AUC values for p-tau217/Aβ40 and p-tau217/Aβ42, which recently received US Food and Drug Administration approval as a blood test for AD using a different assay,^38^ were less than any of the aforementioned tests, and both ratios were elevated in prion disease compared with sAD. P-tau217, p-tau181, and GFAP had no discriminatory value. Illustrative sensitivities and specificities using cutoffs defined by the Youden method^33^ are displayed (Table 2).

In the validation cohort (eTable 2, eFigure 4), similarly to the main study, Lumipulse G NfL/p-tau217 ratio exhibited almost perfect discrimination between sAD and sCJD (AUC 0.986 [95% CI 0.966–1.000], with a greater AUC than NfL (AUC 0.871 [95% CI 0.775–0.968]). In contrast, p-tau217 exhibited some discriminatory value (AUC 0.812 (95% CI 0.706 to 0.917)) with median values higher in sAD than sCJD.

### Cross-Sectional and Longitudinal Analysis of Plasma AD Biomarkers as Markers of Disease Progression in sCJD

**Figure 4:**
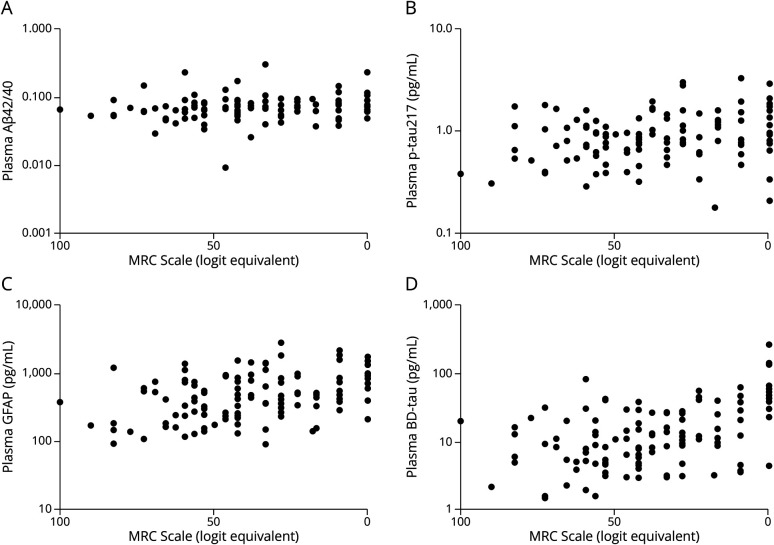
Cross-Sectional Relationship Between Plasma Biomarkers and Disease Stage Measured by MRC Scale (Functional Outcome Measure) in sCJD Cross-sectional dataset using latest available plasma sample for each patient. (A) Age-adjusted Aβ42/40 ratio, (B) p-tau217, (C) age-adjusted GFAP, and (D) BD-tau in plasma plotted against MRC Prion Disease Rating Scale, an interval-level functional outcome measure for CJD running from 100 (no significant impairment) to 0 (bedbound, fully dependent, unable to swallow, and with minimal awareness of surroundings). BD-tau = brain-derived tau; CJD = Creutzfeldt-Jakob disease; GFAP = glial fibrillary acid protein; MRC = Medical Research Council; sCJD = sporadic CJD.

The cross-sectional relationship between plasma biomarker and sCJD disease stage was examined by plotting each individual's biomarker value on a log scale against MRC Scale, a measure of functional ability (Figure 4 and eFigure 5), and days before death (eFigure 6 and eAppendix 2). Using multiple linear regression modeling, with log biomarker as outcome variable, and MRC Scale and *PRNP* codon 129 as explanatory variables, a 1 unit decrease in MRC Scale (more advanced disease stage) was associated with progressively greater increases in log age-adjusted Αβ42/40 (β = −0.00175, 95% CI –0.00330 to −0.000213, *p* = 0.026), log p-tau217 (β = −0.00282, 95% CI –0.00468 to −0.000958, *p* = 0.003), log age-adjusted GFAP (β = −0.00550, 95% CI –0.00801 to −0.00300, *p* < 0.0005), log BD-tau (β = −0.00709, 95% CI –0.0100 to −0.00416, *p* < 0.0005), and log age-adjusted NfL (β = −0.00787, 95% CI –0.0101 to −0.00560, *p* < 0.0005). The regression model for p-tau181 was not better than the null model. There was an independent effect of *PRNP* codon 129 genotype on BD-tau. Relative to codon 129 methionine/valine (MV), higher log BD-tau was associated with methionine/methionine (MM) (β = 0.508, 95% CI 0.341–0.674, *p* < 0.0005) and valine/valine (VV) genotypes (β = 0.198, 95% CI 0.00940–0.386, *p* = 0.0400). Associations between MRC Scale and log (biomarker) are summarized in eTable 3. The results were similar, with no biomarker tested outperforming NfL, when z-score standardized biomarker values were analyzed.

The relationship between biomarker value and rate of sCJD clinical progression was explored by plotting biomarker value on a log scale against each individual's MRC Scale slope, a measure of rate of clinical decline (eFigure 7). In a multiple linear regression model with log biomarker as outcome variable and MRC Scale slope and *PRNP* codon 129 genotype as explanatory variables, there was a positive association between log BD-tau and MRC Scale slope (β = 0.261, 95% CI 0.141–0.381, *p* < 0.0005; eTable 4) with an independent positive association between log BD-tau and codon 129 (VV relative to MV β = 0.197, 95% CI 0.00507–0.389, *p* = 0.044). For all other biomarkers, there was not a significant association between log biomarker and MRC Scale slope (eTable 4). In a subset of cases that underwent molecular strain typing (eTable 5), BD-tau was elevated in MM-1/MM-2 (London classification,^39^ equivalent to Parchi MM1^40^) compared with MM-3 (Parchi MM2) (FC 3.11, IQR 2.13–4.45, Mann-Whitney *U* test, *p* = 0.0084) and in MV-2 (Parchi MV1) compared with MV-3 (Parchi MV2) (FC 2.18, IQR 1.01–8.02, *p* = 0.0298). Within the same *PRNP* codon 129 genotype, the prion strain type associated with more rapid decline exhibited higher BD-tau in both cases.

**Figure 5:**
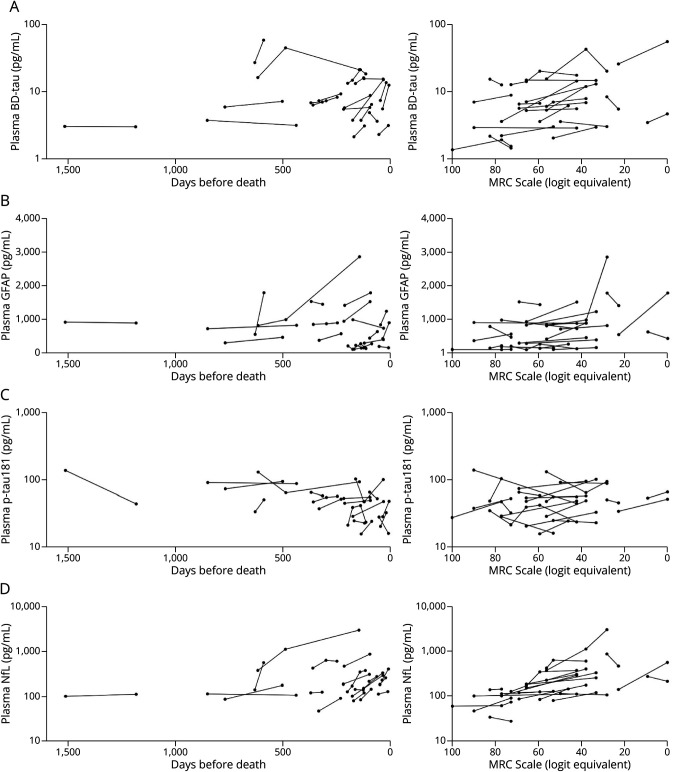
Longitudinal Relationship Between Plasma Biomarkers and Disease Progression in sCJD Longitudinal dataset using serial plasma samples. Each patient's samples are connected by a line. (A) BD-tau, (B) GFAP, (C) P-tau181, and (D) NfL are plotted against measures of disease progression—days before death (left, n = 44 from 20 patients) and MRC Scale (right, n = 49 from 22 patients). Disease progression is from left to right. A summary of the analysis for each biomarker and measure of disease progression is included in eTable 7. BD‐tau = brain‐derived tau; GFAP = glial fibrillary acid protein; MRC = Medical Research Council; NfL = neurofilament light chain; sCJD = sporadic CJD.

In patients with sCJD with serial blood samples, summarized in eTable 6 and eFigure 8, linear mixed effects models were used to explore the longitudinal relationship between change in log_10_(biomarker) and disease progression measured by either MRC Scale or days before death (Figure 5 and eFigure 6). Log NfL increased as patients progressed and MRC Scale decreased (β = −0.00939, 95% CI –0.0128 to −0.00601, *p* < 0.0005), as did, to a lesser extent, log BD-tau (β = −0.00528, 95% CI –0.00831 to −0.00224, *p* = 0.001), and log GFAP (β = −0.00480, 95% CI –0.00819 to −0.00141, *p* = 0.006) but not log p-tau181, log p-tau217, or log Aβ42/40. Only log NfL increased when using decrease in days until death as a measure of progression instead of MRC Scale. Model results are summarized in eTable 7. Analysis of standardized biomarker values produced equivalent findings.

### Classification of Evidence

This study provides Class II evidence that plasma NfL/p-tau217 discriminates patients with CJD from those with AD.

## Discussion

In this study, plasma p-tau217 and p-tau181 measured with Simoa cannot discriminate prion diseases from sAD, because these biomarkers are raised in both conditions. Using our cohort of patients with autopsy-confirmed prion disease, we confirmed this is not simply because of AD copathology. Plasma Aβ42/40 offered only limited discriminatory value. Plasma biomarkers of neurodegeneration (NfL, and to a slightly lesser extent BD-tau) offered much higher discriminatory value, and the NfL/p-tau217 ratio discriminated sAD from prion diseases without significant AD copathology almost perfectly, a finding replicated in a validation study using Lumipulse G assays in a different laboratory on sequentially recruited patients with sCJD and sAD. Our findings indicate that the recent Alzheimer's Association AD diagnostic criteria, in which a single abnormal plasma p-tau217 result is suggested as sufficient to establish a diagnosis of AD,^1^ may result in misclassification of prion diseases as AD. These data suggest a possible role for NfL in blood biomarker diagnostic tests for AD, with NfL/p-tau217 above a cutoff acting as a flag to consider prion disease. Further work might focus on validating our findings prospectively in a clinical setting with expanded comparison groups including other neurodegenerative disorders such as frontotemporal dementia and amyotrophic lateral sclerosis, which may also exhibit an elevated NfL/p-tau217 ratio.^41^

Plasma p-tau217 and p-tau181 have been reported as highly specific diagnostic biomarkers of AD^2,36,37,42,43^; however, in this study, both were elevated across all prion disease diagnostic groups with particularly marked elevation in vCJD cases. Prion disease–related p-tau pathology is known to exist across sporadic, inherited, and acquired prion diseases, but there is a particularly heavy burden in vCJD,^14^ as was the case for vCJD autopsy cases in our study (which also had no ADNC). Previous studies indicate that CSF p-tau181 is elevated in vCJD compared with sCJD,^19^ CSF p-tau181 and p-tau217 are elevated in sCJD compared with nondementia controls,^18,44^ and prion disease–related p-tau pathology correlates with CSF p-tau181.^13^ Collectively this would be in keeping with prion disease–related p-tau pathology driving elevated plasma p-tau181. Further studies with systematic assessment of prion disease–related p-tau pathology and AD-related p-tau pathology are required to confirm this. One study of plasma p-tau181 in prion disease, which used a heterogeneous group of 33 patients with rapidly progressive dementia with 19 sCJD cases, exhibited lower plasma p-tau181 than the AD group, and no elevation compared with controls.^3^ The reasons for the differences between this study and ours are unclear. More in keeping with our current findings, a very recent study reported elevated plasma p-tau217 in CJD compared with healthy controls and similar levels in CJD compared with mild cognitive impairment because of AD.^4^ Lumipulse p-tau217 exhibited some discriminatory value between sAD and sCJD in the validation study (much improved by NfL/p-tau217 ratio) but still below acceptable performance levels for clinical use.^45^ This difference may relate to previously reported greater fold-change of Lumipulse relative to Simoa p-tau217 in AD vs control,^46^ or because the validation cohort used first available sample so the patients with sCJD did not accumulate as much p-tau pathology.

Although plasma Aβ42/40 has some value in discriminating sAD and prion diseases, it performs less well than NfL/p-tau217 ratio, and its 68% sensitivity is below acceptable performance levels.^45^ Better performing plasma Aβ42/40 assays than the Simoa assay have been reported, in particular mass spectrometry–based assays, but the fold change in plasma Aβ42/40 is also small with this technique,^47^ and automated immunoassays similar to the one we used are already validated for clinical use for CSF and are, therefore, closer to application in clinic.^2,48^ The low fold change for plasma Aβ42/40 results in robustness issues, with risk for patient misclassifications.^49,50^ Despite its limitations, it is notable that the Aβ42/40 ratio still has some value in discriminating sAD from prion diseases despite the marked decrease in Aβ42 and Aβ40 in prion diseases relative to sAD and controls.

Plasma biomarkers of disease progression are attractive as noninvasive surrogate endpoints in clinical trials and potentially as aids to prognostication.^16^ In sCJD, cross-sectional analysis reveals that lower MRC Scale, a bespoke functional outcome measure for CJD in which lower scores indicate more advanced disease, has progressively stronger associations with higher Aβ42/40, p-tau217, GFAP, BD-tau, and NfL. In longitudinal analysis, lower MRC Scale showed progressively stronger associations with increases in GFAP, BD-tau, and NfL. Together these data suggest that NfL remains the best plasma biomarker of sCJD disease stage, although BD-tau and GFAP also show promise and may provide insights into different aspects of the underlying disease process.^16,24^ In contrast, BD-tau was the only plasma biomarker associated with rate of clinical decline (MRC Scale slope). In this regression model, there were independent associations between BD-tau and MRC Scale Slope, and BD-tau and *PRNP* codon 129 genotype (a known predictor of rate of clinical decline in sCJD). Within *PRNP* codon 129 genotypes MM and MV, BD-tau was elevated in the PrP^Sc^ molecular strain type associated with more rapid decline. Collectively, this suggests a potential role for BD-tau as a biomarker of neurodegeneration intensity and in predicting rate of clinical decline, with the desirable property of providing independent information on rate of clinical decline to *PRNP* codon 129 genotype. In contrast to previous studies,^3,30^ BD-tau was not elevated in sAD relative to controls.

Limitations of this study include lack of testing of healthy controls for undisclosed neurologic disease; longitudinal analysis with serial plasma sampling only being available in a subset of patients; small numbers of some less common diagnostic groups; and lack of data on renal function, which can affect biomarker levels.

This study reports poor performance of plasma p-tau217 and p-tau181 in discriminating sAD from prion diseases and a solution: the NfL/p-tau ratio. It also identifies BD-tau as a biomarker strongly associated with rate of decline in sCJD and supports NfL as the strongest plasma biomarker of disease stage in sCJD.

## Glossary

AD: Alzheimer disease
ADNC: AD neuropathologic change
AUC: area under the curve
BD-tau: brain-derived tau
FC: fold change
GFAP: glial fibrillary acid protein
iAD: iatrogenic AD
iCJD: iatrogenic Creutzfeldt-Jakob disease
IPD: inherited prion disease
IQR: interquartile range
NfL: neurofilament light chain
NHNN: National Hospital for Neurology and Neurosurgery
PRNP: prion protein gene
ROC: receiver operating characteristic
sAD: sporadic AD
sCJD: sporadic CJD
vCJD: variant CJD
